# Material properties of human vertebral trabecular bone under compression can be predicted based on quantitative computed tomography

**DOI:** 10.1186/s12891-021-04571-4

**Published:** 2021-08-18

**Authors:** Dominic Gehweiler, Marius Schultz, Martin Schulze, Oliver Riesenbeck, Dirk Wähnert, Michael J. Raschke

**Affiliations:** 1 AO Research Institute Davos, Clavadelerstrasse 8, 7270 Davos, Switzerland; 2 University Hospital Muenster, Department of Trauma, Hand and Reconstructive Surgery, Albert-Schweitzer-Campus 1, Building W1, 48149 Muenster, Germany; 3 Department of Orthopedics, University Hospital Muenster, Albert-Schweitzer-Campus 1, 48149 Muenster, Germany; 4 Department of Trauma Surgery and Orthopedics, Protestant Hospital of Bethel Foundation, University Hospital OWL of Bielefeld University, Campus Bielefeld-Bethel, Burgsteig 13, 33627 Bielefeld, Germany

**Keywords:** Biomechanics, Human vertebra, Cancellous bone, Material properties

## Abstract

**Background:**

The prediction of the stability of bones is becoming increasingly important. Especially osteoporotic vertebral body fractures are a growing problem and an increasing burden on the health system. Therefore, the aim of this study was to provide the best possible description of the relationship between the material properties of human vertebral trabecular bone measured under the most physiological conditions possible and the bone mineral density (BMD) determined by clinical quantitative computed tomography (QCT).

**Methods:**

Forty eight cylindric cancellous bone samples with a diameter of 7.2 mm obtained from 13 human fresh-frozen lumbar vertebrae from 5 donors (3 men, 2 women) have been used for this study. After the specimens were temporarily reinserted into the vertebral body, the QCT was performed. For mechanical testing, the samples were embedded in a load-free manner using polymethylmetacrylate (PMMA). The surrounding test chamber was filled with phosphate buffered saline (PBS) and heated to 37 °C during the test. After 10 preconditioning load cycles, destructive testing was performed under axial compression. After determining the fracture site, BMD has been evaluated in this region only. Regression analyses have been performed.

**Results:**

Fracture site had an average length of 2.4 (±1.4) mm and a position of 43.9 (±10.9) percent of the measurement length from the cranial end. No fracture reached the embedding. The average BMD at the fracture site was 80.2 (±28.7 | min. 14.5 | max. 137.8) mgCaHA/ml.

In summary the results of the regression analyses showed for all three parameters a very good quality of fit by a power regression.

**Conclusion:**

The results of this study show that QCT-based bone density measurements have a good predictive power for the material properties of the vertebral cancellous bone measured under near to physiological conditions. The mechanical bone properties of vertebral cancellous bone could be modelled with high accuracy in the investigated bone density range.

## Background

The prediction of the mechanical integrity of bones is becoming increasingly important. Especially in an ageing society, osteoporotic vertebral body fractures are a growing problem and an increasing burden on the health system [1, 2]. A valid clinical method for predicting fracture probabilities and bone strength could help to address this problem and allow the development of an appropriate strategy to prevent osteoporotic fractures or implant failure.

However, valid computational models of the human spine are required to transfer in vitro material properties into clinical applicability. Therefore, biomechanical material properties are indispensable for making predictions about the mechanical integrity of the vertebral bone. They can be measured in vitro, but not non-invasively in vivo.

However, quantitative computed tomography (QCT) allows precise measurement of bone mineral density (BMD) [3], and can be determined with virtually any clinical CT. It is suitable as a ubiquitously available tool for non-invasive determination of BMD in patients and is therefore useful for non-invasive characterization of bone material properties. Several publications have already described the correlations between bone material properties and BMD [4–7], but do not reach a consistent conclusion due to relevant differences in methodology [8].

The accuracy of a model depends on the goodness of fit of the formulas describing the relationship between clinically measured image data (BMD) and in vitro measured material properties. In this context, the quality of the in vitro tests is decisive [8, 9]; here, special care must be taken to eliminate as many confounding variables as possible.

Regarding the characterization of material properties of vertebrae, there are numerous studies that tested cancellous bone [5–7, 9–13], but only four of them used methods to prevent end artifacts [6, 7, 9, 11] and QCT data are available in only two studies [6, 12].

The aim of this study was to provide the best possible description of the relationship between the BMD determined by QCT and the material properties of human vertebral trabecular bone under the consideration of minimization of influencing variables in relation to the most comparable study of Kopperdahl et al. [6]. Therefore, the specimens were scanned with surrounding vertebral body evacuated in a water bath to mimic the in vivo situation, avoid large density gradients and minimize the associated effects (e.g. beam hardening and partial-volume effects) which would lead to deviant BMD measurements not comparable to clinical application. Finally, the BMD was evaluated in the fracture zone of each specimen. This procedure allows the correlation of the mechanical failure and BMD at the fracture location. Regarding mechanical testing we used polymethylmethacrylate (PMMA) for cranial and caudal specimen embedding to avoid end artefacts. Additionally, mechanical testing was performed in phosphate buffered saline (PBS) at a temperature of 37 °C to mimic physiologic conditions.

## Methods

Thirteen human lumbar vertebrae (in the range of L1 to L5) from 5 body donors with a mean donor age of 58.4 (±4.5) years, provided by Science Care (*Phoenix Arizona, USA*), were selected. The gender distribution was three women to two men. To rule out preexisting fractures, bony lesions or diseases, the medical record was reviewed and radiographs in 2 planes were obtained.

### Sample preparation

Mechanical bone properties can be influenced by storage and processing methods [14]. Although thawing and re-freezing does not appear to produce any significant change in mechanical properties [14], it has been avoided whenever possible. Therefore, the frozen lumbar spines were sawed through the intervertebral discs without damaging the vertebral bodies. Subsequently, if possible, four samples with a diameter of 7.2 mm were taken axially from the frozen vertebral bodies by means of a water-cooled diamond-coated hollow drill (KARL STORZ GmbH & Co. KG, Tuttlingen, Germany). This procedure ensured that a clean cut was achieved without damaging the adjacent trabeculae and that the bone marrow was not washed out. Due to the geometry of L5, only 3 samples could be obtained there twice and 2 once. In total 48 samples could be obtained.

### QCT

**Fig. 1:**
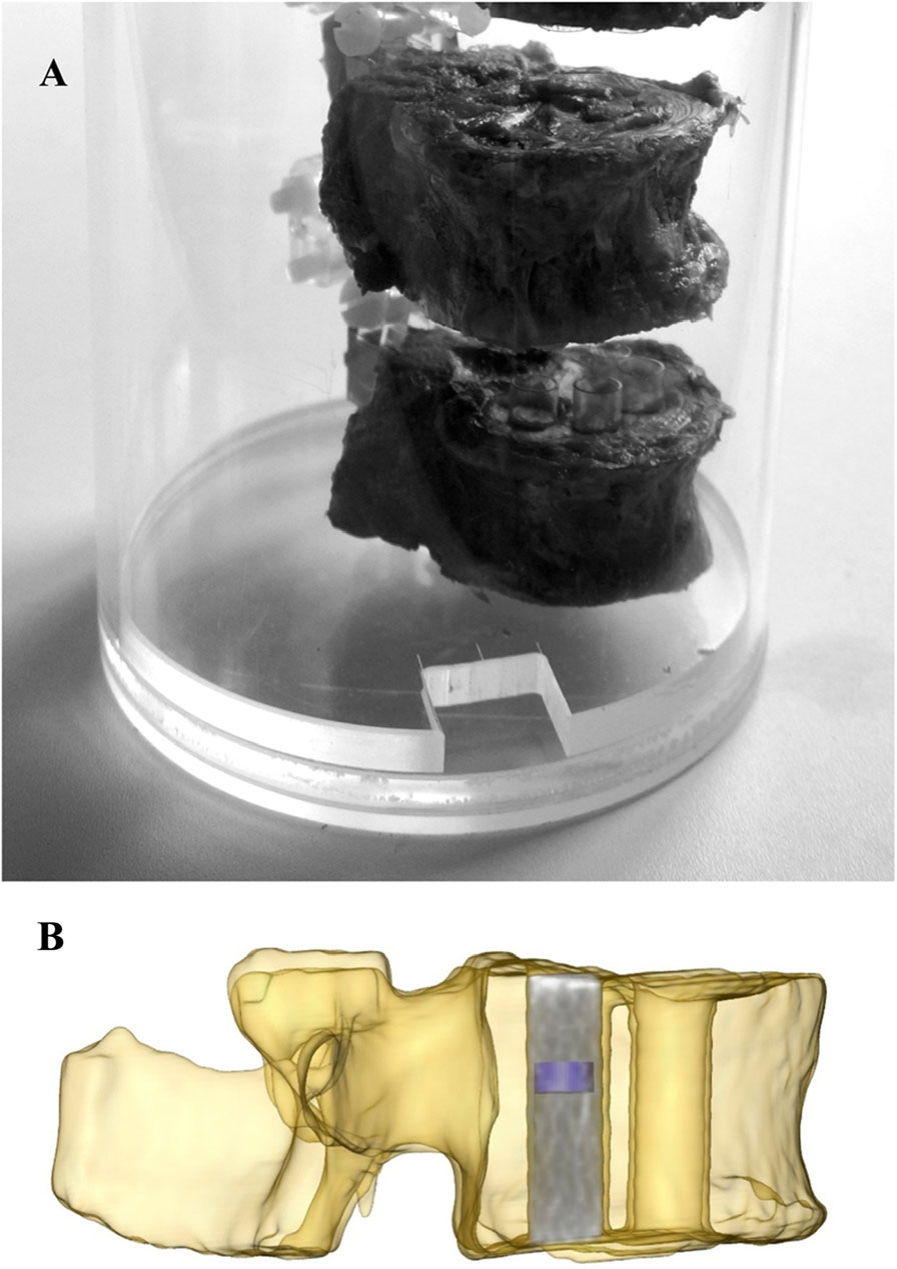
**A** Picture showing the fixation of bone cylinders within their original positions in the vertebral bodies with a small acrylic glass tube at the end plates. After axially aligned fixation of all vertebrae of one donor in the acrylic glass cylinder, it was filled with water and evacuated for standardized QCT scans. **B** Picture showing the BMD assessment in the QCT within the fracture zone (blue ROI) of a bone cylinder visualized in a 3D surface model of a vertebra

For the QCT measurements, the single samples were temporarily fixed at their original positions in the vertebral bodies with a small acrylic glass (polymethylmethacrylate, PMMA) tube at the end plates. After axially aligned fixation of all vertebrae of one donor in an acrylic glass cylinder, it was filled with water and evacuated after complete thawing of the vertebrae (see Fig. 1A). This approach provided comparability with clinical QCT measurements and ensured that no artifacts were produced on the surface of the samples and that the BMD measurement was not negatively affected by small air bubbles inside the bone.

All QCT scans were performed with a SOMATOM Definition (Siemens Healthcare GmbH, Erlangen, Germany) with a slice thickness of 0.6 mm. Since sharp kernels lead to an overestimation of BMD [15], soft kernels were chosen (B30s, D30f) in this study. The tube voltage has no relevant influence when using a BMD calibration phantom [16]. In this study the tube voltage was set to 140kVp and the Siemens BMD calibration phantom [3] was chosen.

### Mechanical testing

The individual samples were placed in the refrigerator for slow defrosting the evening before mechanical testing. Three hours before the start of the test, they were placed at room temperature in a phosphate-buffered saline solution (PBS) to create a physiological test environment. The samples were kept moist with PBS for the entire duration of the experiment to avoid drying out.

If a bone is compressed between two parallel plates, so-called end artifacts occur which lead to an underestimation of the modulus of elasticity [9, 17]. To avoid end artifacts during compression testing, the ends of all specimens were embedded in a polymethylmethacrylate (PMMA, Technovit 3040®, Heraeus Kulzer GmbH, Wehrheim, Deutschland).

**Fig. 2:**
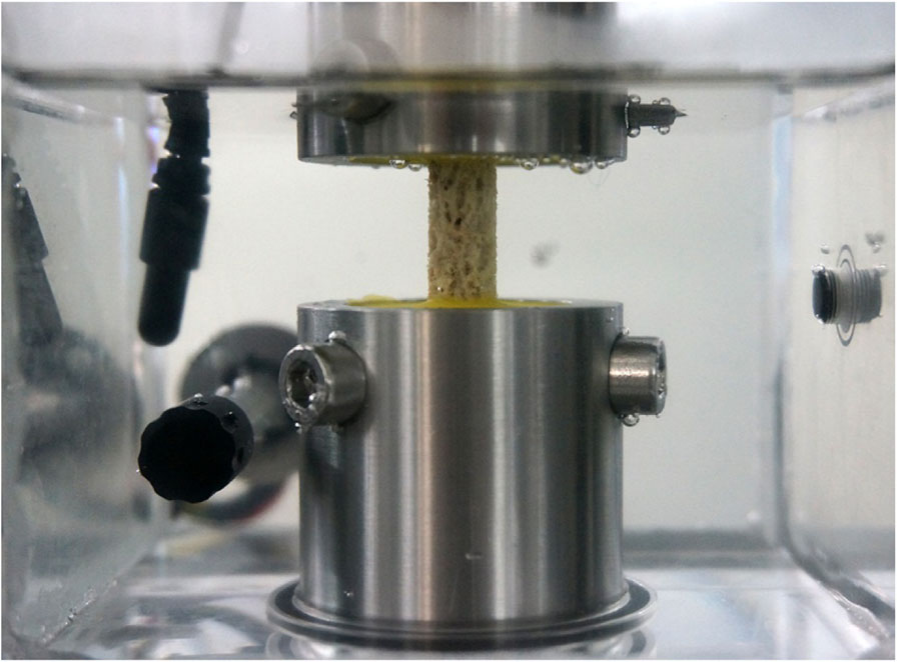
Test set-up of an embedded sample, clamped force and torque free on the materials testing machine, in the test chamber, filled with 37 °C warm PBS, ready for testing

A custom-made holder was manufactured for embedding the first side of the sample over a defined length exactly centered in the potting form. After curing, the specimen was removed from the embedding mould and the PMMA block was clamped in the actuator of a spindle-driven material testing machine (Zwick/Roell Z005 (Zwick GmbH & Co. KG, Ulm, Germany)). For embedding of the second side, PMMA was filled into the lower holder and the sample was lowered with the test machine until the distance between the two PMMA parts was 14 mm. The load cell of the materials testing machine was then zeroed and the material testing machine was programmed to keep the measured force to 0 N in order to compensate for the minimum size change occurring during the PMMA polymerization process. This procedure ensured that the specimens were clamped without any forces or torques exactly axially in the material testing machine. Subsequently, the surrounding test chamber was filled with PBS, heated to 37 °C and kept at 37 °C during the test (see Fig. 2).

Before destructive testing, a preconditioning with ten cycles of − 0.175% (tension) to 0.35% (compression) strain was performed [18] to achieve a correct zero crossing. According to Linde and Hvid, a low strain rate should be used to determine the modulus of elasticity of bone [19]. The test speed for cyclic loading and destructive testing was therefore set to a change in length of 0.083%/s, analogous to Chevalier et al. [18]. The termination criterion was a force drop compared to the maximum force of 30% or a length reduction of 2 mm. After unclamping the specimen, the fracture position was measured with a caliper gauge.

### Data evaluation

**Fig. 3:**
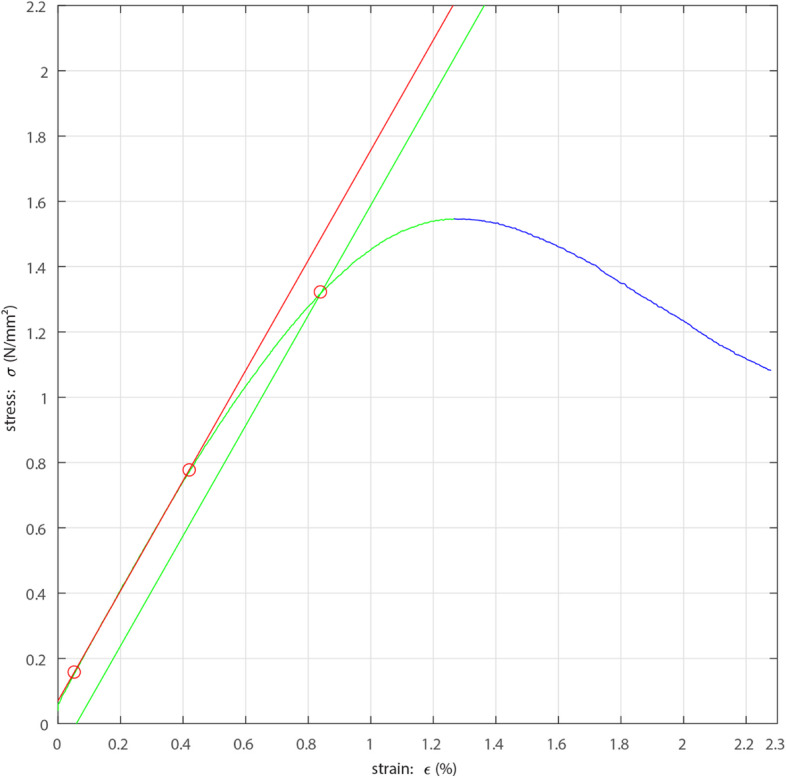
Exemplary output of an evaluation: elastic modulus = slope of the red line determined by linear regression between 10 and 50% of maximum stress, yield offset = green line, yield stress = intersection of the green curve with the green line, maximum stress = apex of the stress-strain curve (transition from green to blue curve)

Recorded machine data was imported to MATLAB (Version R2014b, MathWorks, Natick, USA). The yield offset was set to 0.1% according to the recommendations of Keller et al. [20]. The following parameters were automatically determined for each sample using a custom-made MATLAB script: elastic modulus, yield stress and maximum stress; the elastic modulus was determined by linear regression in the initial linear portion of the stress-strain curve (see Fig. 3).

The mean BMD within a cylindrical ROI of 5 mm diameter was measured after converting Hounsfield units (HU) to volumetric BMD (vBMD) values. The space of 1 mm between the ROI and the edge of the sample was chosen to reduce the influence of potential artifacts. Since the fracture region of the samples in the compression test was on average 2–3 mm in height, the ROI thickness was set to 3 mm for all samples, to measure the BMD only at the fracture region (Fig. 1B).

The regression models including the calculation of the respective goodness of fit were created in R (Version 3.3.1, R Core Team, Vienna, Austria). Polynomial regression to the fourth degree (linear, quadratic, cubic, biquadratic), as well as exponential and logarithmic regression and a combination of these were selected as potential regression types. The model evaluation was based on the Akaike Information Criterion (AIC) [21], which enabled a meaningful pre-selection of the possible model combinations and avoided a potential over-fitting. For the models with the three lowest AIC values, the quality of fit was determined.

## Results

The fracture site had an average length of 2.4 (±1.4) mm and a position of 43.9 (±10.9) percent of the measurement length from the cranial end. All fractures occurred in the purely bony area without affecting parts of the in PMMA embedded bone. One sample had to be excluded due to insufficient embedding. With two further specimens, no unambiguous quasi-linear part in the stress strain diagram for determining the modulus of elasticity and no clear fracture point could be determined, so that these specimens were also excluded. The average BMD at the fracture site was 80.2 (±28.7 | min. 14.5 | max. 137.8) mgCaHA/ml.

**Fig. 4:**
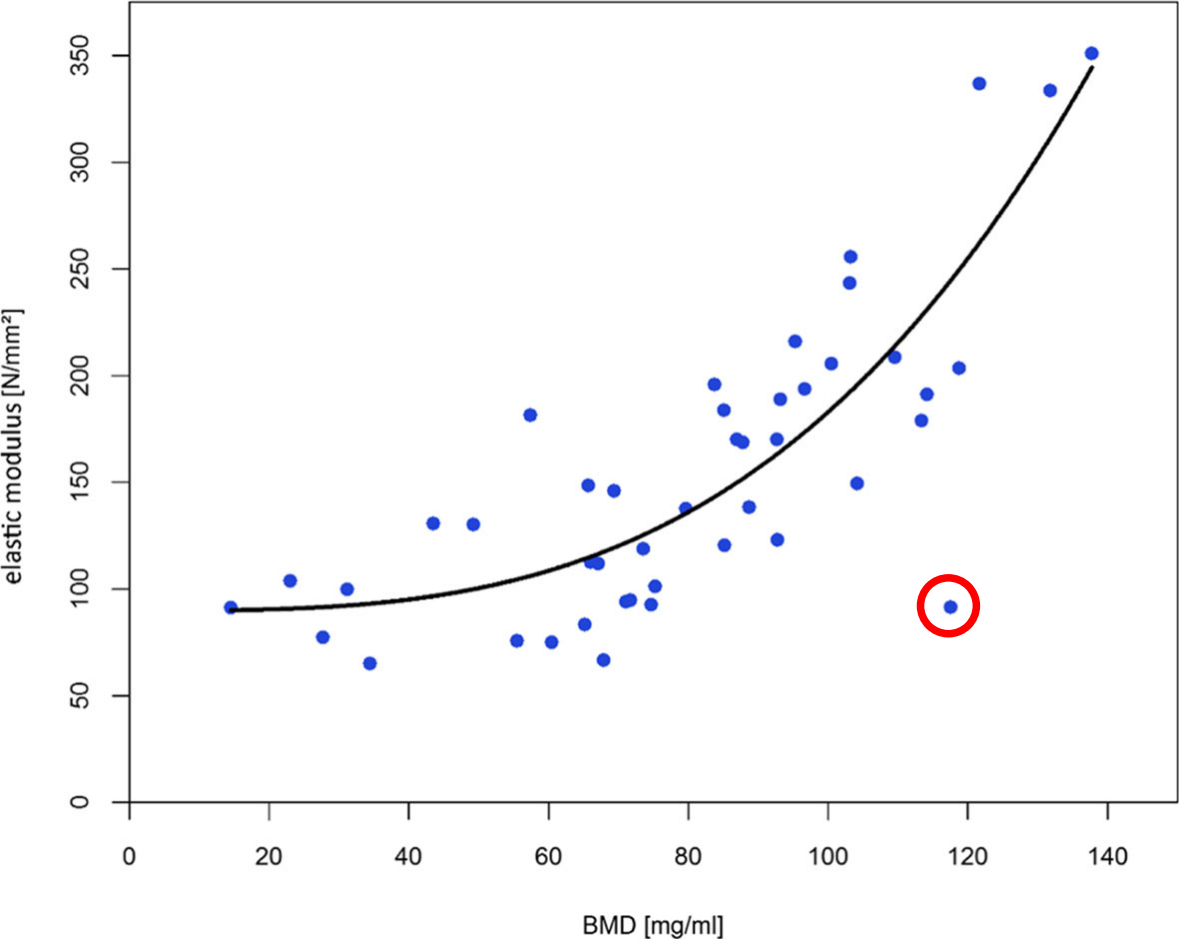
Power regression for elastic modulus (R^2^ = 0.65)

**Fig. 5:**
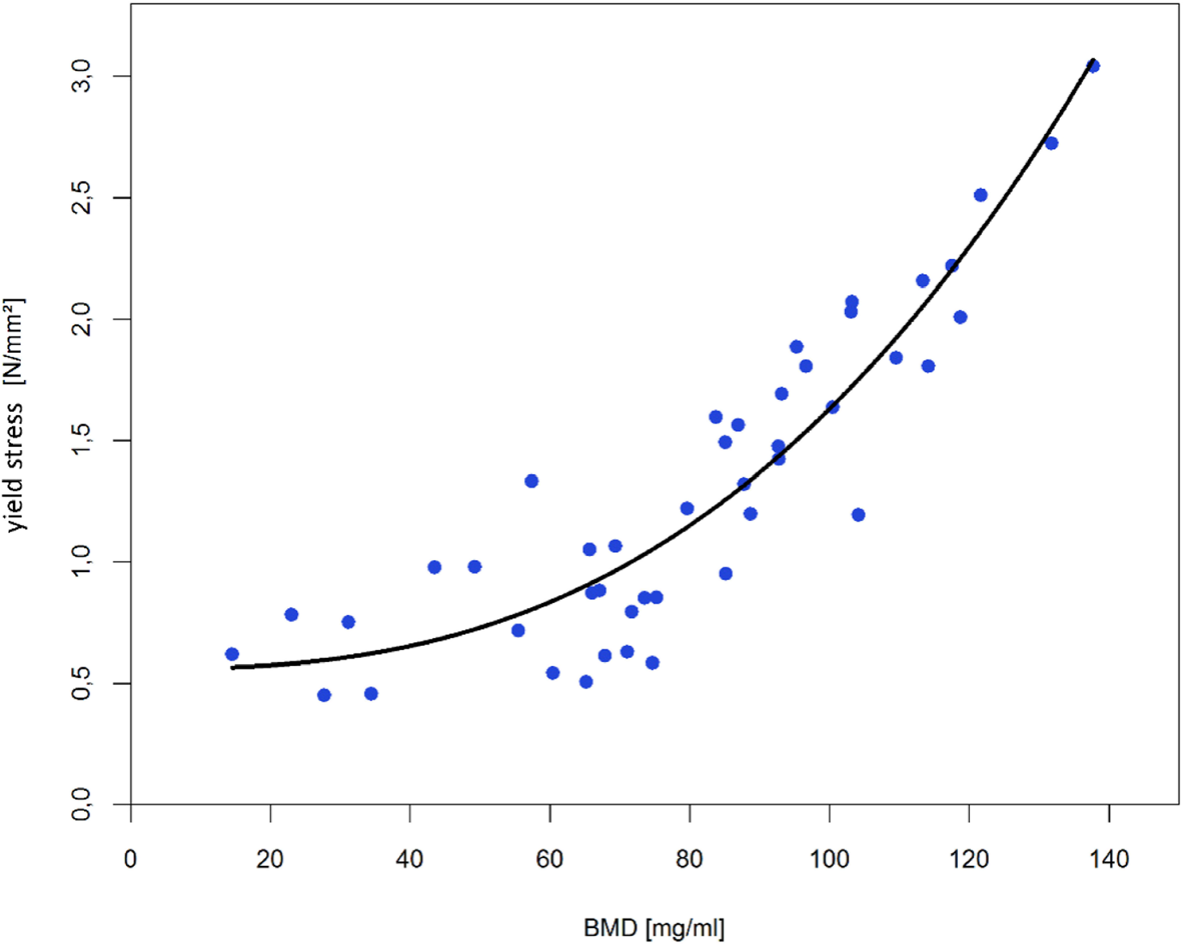
Power regression for yield stress (R^2^ = 0.85)

**Fig. 6:**
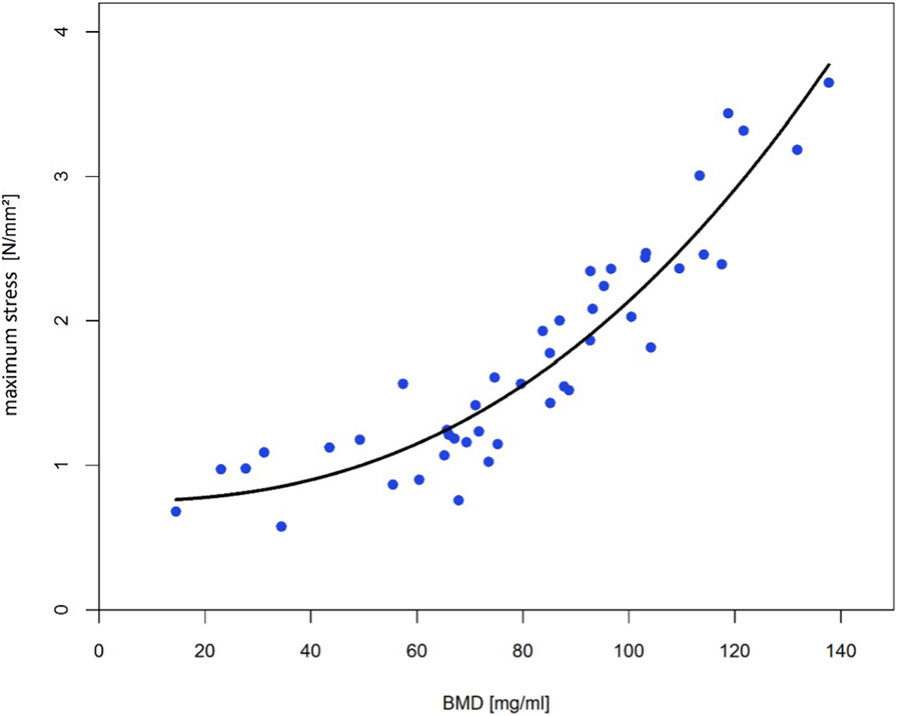
Power regression for maximum stress (R^2^ = 0.88)

In summary the results of the regression analyses showed for all three parameters a very good quality of fit by a power regression (see Table 1 and Figs. 4, 5 and 6).

**Table 1:** Overview of the regression models and determination coefficients

Parameter	Model	R^2^
elastic modulus	*E* = 89.87 + 4.81*e*^−5^ ∗ *BMD*^3.14^	0.65
yield stress	*Y* = 0.56 + 5.08*e*^−6^ ∗ *BMD*^2.66^	0.85
maximum stress	M = 0.75 + 1.86*e*^−5^ ∗ *BMD*^2.44^	0.88

If the data point marked red in Figure 4 would be considered as an outlier and therefore not considered for the regression, the following formula (*E* = 88.55 + 5.36*e*^−5^ ∗ *BMD*^3.13^) results with a considerably better coefficient of determination (R^2^ = 0.76).

## Discussion

Morgan et al. were able to show that the correlation between bone density and elastic modulus is dependent on anatomical positions and the prediction error can rise above 60% if this distinction is ignored [7]. For this reason, the present study will refrain from comparisons with other anatomical localizations. In the literature there are several studies dealing with the material properties of the vertebrae. However, only the study by Kopperdahl et al. [6] shows several parallels to our work; a cylindrical sample geometry with preserved bone marrow was chosen, prior to QCT measurement the vertebrae were evacuated and end artifacts were prevented by the use of brass end caps. The preservation of the bone marrow in the samples is reported to have no influence on the measured material properties under physiological loading [20, 22]. However, Wolfram et al. showed that the difference in Young’s modulus between wet and dry bone specimens alone yielded 29% lower values [23].

The decisive differences with study by Kopperdahl et al. are that, firstly, the mechanical test was not carried out at 37 °C. However, Turner and Burr were able to show that a measurement at room temperature leads to a measurable error with an increased modulus of elasticity (2 to 4%). The influence of temperature is even more pronounced in the failure test. Samples tested at room temperature underwent twice as many cycles to failure as those tested at 37 °C [24]. For biomechanical tests, therefore, measurement at 37 °C ambient temperature was recommended [24]. It could also be demonstrated by Alegre et al. that reduced temperature leads to changes in the mechanical properties of collagen, resulting in a change in stiffness (+ 25%) and strain (− 9%) [25].

Second, the QCT examination of the cancellous bone samples was performed without the surrounding vertebral bodies. In our experiments, however, the samples were measured in their bony environment in order to achieve as realistic conditions as possible and minimize artifacts that are unavoidable in regions with large density gradients. Thirdly, the bone density was not determined exactly at the fracture position. A deviation of the ROI position in randomly selected cancellous bone specimens from our test series by a few millimeters resulted in a change in HU measurement values of up to more than 30%. The determination in the center of the sample or the use of the mean bone density throughout the entire sample therefore does not sufficiently take into account the variability of bone density within a cancellous bone sample. This could result in inaccurate computational models and therefore, we recommend the use of the BMD at the fracture region. Our approach was based on the assumption that the fracture will occur in the weakest area of an inhomogeneous specimen. Therefore, it is only consequent to try to determine the density of exactly this area.

Comparing the results, Kopperdahl et al. presented comparable measured values for the yield stress, where the Young’s modulus values are significantly higher than in the present study. They argued that as a result of testing specimens between end caps to minimize end artifact errors, their mean modulus was 4.8–14 times higher than in previously reported studies [6]. The situation is different in the low BMD range up to 50 mgCaHA/ml. Here, the values measured in our study are higher for both Young’s modulus and yield stress. Assuming that bone density decreases from the end plates towards the center of the vertebral body, this phenomenon can be well explained by the different methods of measuring bone density. While Kopperdahl et al. determined the BMD of the entire sample, in the present study BMD was determined only at the weakest point where the fracture occurred. However, this procedure may increase the reliability of the measured parameters and can be seen as a unique approach of the present study. Furthermore, it would have been interesting to compare the not reported maximum stress of Kopperdahl et al. with the present values.

The maximum stress of the bone samples in our study was on average 32% higher than the yield stress. In contrast, Hansson et al. report a difference of 12% [10]. This could possibly be due to the choice of yield offset, which was 0.1% lower in our study. However, a yield offset of 0.1% corresponds to the value recommended by Keller for mechanical testing of bones [5]. He states, that if choosing the 0.2% offset criterion “due to the slightly different shape of stress-strain curves of weak specimens compared with stronger specimens, a yield strain close to and even larger than the ultimate strain is sometimes found in weak specimens” [5], which could be observed in some of this study’s samples as well.

The limitations of the present study are the limited BMD range of available samples, the limited number of donors and the multiple sampling of donors. Therefore, the definition range (D) of the above models corresponds to the bone density range investigated (14.54–137.75 mgCaHA/ml). Extrapolation beyond the minimum or maximum value is not recommended. On the other hand, the low bone density range is of particular interest in the presence of osteopenia or osteoporosis, which is why donors with a high donor age were deliberately used.

## Conclusion

The results of this study show that QCT-based bone mineral density measurements have a good predictive power for the material properties of the vertebral cancellous bone measured under optimized conditions: BMD acquisition using clinical QCT and scanning with surrounding bone evacuated in water bath, BMD evaluation at fracture region, mechanical testing in 37 °C warm PBS with embedding to prevent end artefacts. The mechanical bone properties - expressed as modulus of elasticity, yield stress and maximum stress of vertebral cancellous bone could be modelled with high accuracy in the investigated bone mineral density range. In the future, experiments with non-axial loads and taking into account other bony structures of the spinal column should follow. Finally, in order to be able to assess the individual risk of a patient’s vertebral body fracture, further investigations of the overall stability of the spinal column by the supporting apparatus and the influence of individual clinical factors are also necessary.

## Data Availability

The datasets used and analysed during the current study are available from the corresponding author on reasonable request.
